# Sustained delivery of functional vascular endothelial growth factor from nanoporous silica nanoparticles into a fibrin gel

**DOI:** 10.1371/journal.pone.0326561

**Published:** 2025-06-26

**Authors:** Karen Besecke, Sarah Zippusch, Florian Helms, Ulrike Böer, Mathias Wilhelmi, Peter Behrens, Nina Ehlert

**Affiliations:** 1 Institute of Inorganic Chemistry, Leibniz University Hannover, Hannover, Germany; 2 NIFE – Lower Saxony Center for Biomedical Engineering, Implant Research and Development, Hannover, Germany; 3 Department of Cardiothoracic-, Transplantation, and Vascular Surgery, Hannover Medical School, Hannover, Germany; 4 Department of Vascular- and Endovascular Surgery, St. Bernward Hospital, Hildesheim, Germany; BRAC University, BANGLADESH

## Abstract

The unsolved major issue of large-scale tissue engineering scaffolds is insufficient initial vascularization. The lack of oxygen and nutrient supply for migrating cells inevitably leads to cell death and subsequently to implant failure. One approach to resolve this problem is the development of pre-vascularized tissue constructs. Following this idea, the installation of micro-vessels within a fibrin scaffold is a promising approach. To induce the formation of these micro-vessels a long-term release of vascular endothelial growth factor (VEGF), which is a key player in endothelial growth, is necessary. As drug carriers, nanoporous silica nanoparticles (NPSNPs) were chosen and loaded with VEGF by the interaction with grafted amino groups. Physicochemical characterization (TEM, XRD, N_2_ physisorption, zeta potential, and thermogravimetric analysis) revealed nanoparticles with approximately 44 nm in diameter, pore sizes of 3 nm, and a successful amino modification of the surface. The recorded release profiles showed a possible long-term release of up to 100 days. With adapted loading concentrations for *in vitro* testing, the released doses were applied within an *in vitro* 2D sandwich tube assay with endothelial cells and compared to repeatedly given doses of VEGF in solution. The VEGF released from the NPSNPs led to an equal tube formation, which qualifies them as an effective tool for VEGF delivery inside large-scale tissue constructs that cannot be reached from the outside.

## Introduction

The research in the field of tissue engineering, which has the aim to restore biological tissues by synthetic tissue constructs, is rapidly progressing due to the high clinical demand for tissue replacement materials. However, cells in deeper layers of avascular bioartificial tissue constructs may not be supplied quickly enough with oxygen and nutrients, which bears the risk of cell death and eventually even implant failure, thereby limiting the clinical application of large-scale bioengineered tissue constructs until today. A promising approach to overcome this problem aims to generate pre-vascularized bioartificial tissues, that means artificial tissue mimicking materials with integrated cells and pre-formed blood channels for nutrient supply. In recent studies, several strategies for pre-vascularization have been established and applied. Methods for pre-vascularization include 1) scaffold functionalization, e.g., by growth factor delivery, 2) scaffold engineering, e.g., by formation of microchannels, molecular gradients, or micropatterning, 3) cell-based techniques, e.g., co-cultures with endothelial cells or application of growth factor-producing cells [1,2].

As a potential component for the pre-vascularized tissue construct, we chose fibrin. Fibrin is a versatile biomaterial applied for various tissue engineering approaches [3]. Advantageous characteristics of fibrin are its natural pro-angiogenic properties [4] (support of the formation of new blood vessels from pre-existing ones), its high number of various cell binding sites, and its biodegradability [5]. The fact that it is already used in clinical applications as sealant [6], also in cardiovascular surgery [7], makes it particularly attractive as a scaffold material for tissue constructs. Furthermore, its stiffness can be adjusted in direct relation to the fibrinogen concentration used. However, it has to be considered that it has a generally rather low biomechanical stability [8].

To support the formation of endothelial tubes within the fibrin scaffold (mechanically pre-formed channels in the fibrin scaffold that were coated with endothelial cells), vascular endothelial growth factor (VEGF) was applied as it is known to be the most important growth factor for (pre-)vascularization and an essential parameter for increasing the permeability of blood vessels and the induction of vascularization [9]. VEGF influences the behavior of endothelial cells by inducing cell division as a mitogen and by promoting cell migration. Furthermore, it modulates the gene expression pattern of endothelial cells and their morphology and can therefore be regarded as a survival factor [9,10].

However, if VEGF is applied in uncontrolled high doses, negative effects like excessive vascular proliferation and the formation of aberrant vessels and vascular tumors can appear [11–13]. These investigations reveal that VEGF has a strong or even critical impact on the vascular system and should therefore be delivered in a quantitatively, spatially, and temporally controlled manner [14]. Following these requirements, various VEGF delivery systems have already been developed and reviewed by Beheshtizadeh *et al.* [15] As carrier systems either inorganic (e.g., silica, hydroxyapatite, quantum dots) or organic (e.g., natural polymers like chitosan or alginate, synthetic polymers like polylactid-co-glycolide (PLGA) or polyethylene glycol (PEG)) carriers are applied. Further possible systems are liposomal or microfluidic systems. The immobilization of VEGF in most of these systems is based on the principles of physical adsorption or encapsulation. A collection of particle- and matrix-based drug delivery systems, and the combination of both is presented in the electronic supplementary information (ESI) (S1 Table in S1 File).

Nanoporous silica (non-crystalline silicon dioxide) nanoparticles (NPSNPs) are not only widely used as drug delivery, but also as monitoring vehicles and are promising candidates to combine diagnosis and therapy in one [16]. They can easily be combined with (in)organic material to be used for applications such as magnetic resonance imaging (MRI) and magnetic particle imaging. NPSNPs are tunable and robust, their release mechanisms, which are generally based on diffusion, can be adjusted by either change in pore sizes, loading concentration of desired drug or molecule, or by creating stimuli-responsive NPSNPs, which only start the drug release upon certain stimulating changes in their close environment [16]. In the present study, we used NPSNPs as drug delivery vehicles because they already proved their capability as sustained protein drug delivery systems for example for the brain-derived neurotrophic factor (BDNF) and bone morphogenetic protein 2 (BMP2) [17,18]. NPSNPs are biocompatible, have a high inner surface area and pore volume, and can be easily chemically modified due to present silanol groups (-Si-OH) [19,20] which is essential for the installation of attractive interactions between protein and particle surface.

**Fig 1:**
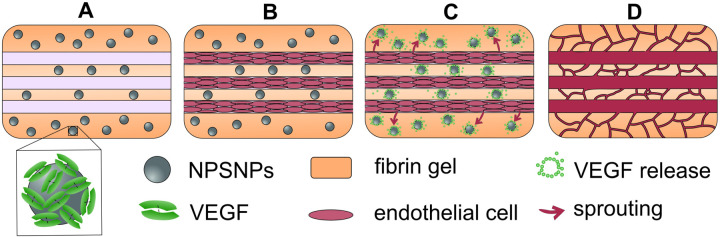
Schematic: step-wise generation of a pre-vascularized fibrin implant. A) 3-Dimensional fibrin matrix with preformed channels including VEGF-loaded NPSNPs, molding described in detail by Helms *et al.* [21], B) endothelialization of the pre-formed channels; C) sprouting of the endothelial cells into the fibrin scaffold, supported by the VEGF released from the NPSNPs; D) final pre-vascularized implant including a multi-scale vascular network.

The aim of this study was to achieve a long-term release of VEGF in a bioartificial fibrin-based scaffold to induce the guided ingrowth of endothelial cells by a VEGF gradient [21]. For the pre-vascularization of bioartificial constructs, a multi-scale vascular network is needed. A VEGF gradient installed by VEGF release from NPSNPs embedded in the fibrin matrix can then potentially induce directed capillary sprouting from pre-formed endothelialized channels and thus, enable the generation of a true multi-scale vascular network in large-scale fibrin base bioartificial tissues (Fig 1).

## Experimental

### Materials

All chemicals and cell culture reagents were purchased from commercial sources and were used without further purification. (3-Aminopropyl) triethoxysilane (APTES), bovine serum albumin (BSA), cetyltrimethylammonium bromide (CTAB), diethanolamine (DEA), phosphate-buffered saline (PBS, Dulbecco’s L1820), tetraethyl orthosilicate (TEOS), 10X medium 199 (10X M199), and calcium chloride were purchased from Sigma-Aldrich (München, Steinheim, Germany). Absolute ethanol (EtOH) and sodium hydroxide solution were purchased from Merck (Darmstadt, Germany). VEGF_165_-A was acquired from PeproTech (Hamburg, Germany) as a sterile-filtered and lyophilized product. According to the manufacturer it was produced in *Escherichia coli* (*E. coli*) and had a purity of ≥98%. VEGF_165_-A was quantified via an enzyme-linked immunosorbent assay (ELISA) kit from PeproTech (Human VEGF_165_ Standard TMB ELISA Development Kit) in combination with a buffer kit from the same company. Thrombin (Beriplast®, CSL Behring, Marburg, Germany) was commercially acquired and fibrinogen was isolated from plasma obtained from the Institute for Transfusion Medicine of the Hannover Medical School (MHH). Fibrinogen was isolated from human fresh frozen plasma obtained from Hannover Medical School blood bank from healthy donors after informed consent. Briefly, plasma was frozen at −80°C for at least 24 h. At least 24 h before isolation of precipitated fibrinogen, plasma was transferred to 2–7°C. Fibrinogen was subsequently cryoprecipitated at 4°C by centrifugation at 3,000 x *g* for 40 min. Pellets were overlayed with ice-cold ultrapure water. Fibrinogen concentration was determined using the Clauss method based on the clotting time [22] (Hematology of Hannover Medical School). Aliquots were stored at −20°C.

### Synthesis and surface modification of NPSNPs

**Fig 2:**
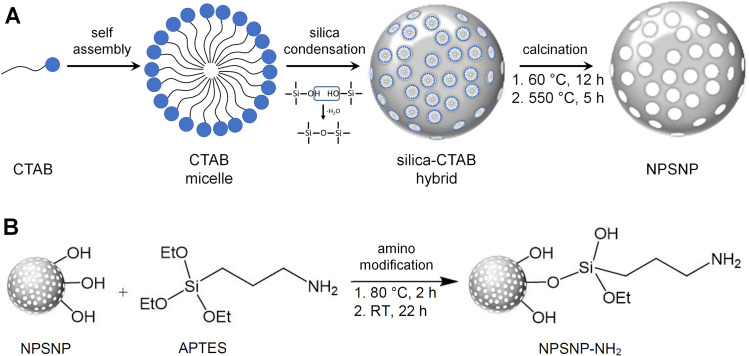
Silica nanoparticle synthesis and modification. A) The structure-directing agent CTAB forms micelles by self-assembly. Silica condensation takes place around these micelles, forming a silica-CTAB hybrid. After calcination, nanoporous silica nanoparticles are produced. B) Amino modification is carried out via the reaction of silanol groups with APTES.

NPSNPs were prepared after a modified synthesis of Qiao *et al.* [23] previously published by our group (Fig 2A) [17]. First, 3.16 g CTAB and 0.23 g DEA were given to a solution of 75.0 mL ultrapure water and 13.4 mL absolute ethanol. While stirring the mixture was heated up to 40°C and after 30 min, 8.56 mL TEOS were added. Then, the reaction mixture was stirred for additional 2 h. The formed nanoparticles were centrifuged (30 min at 18 000 g), washed twice with water and once with ethanol, and dried overnight at 60°C. Finally, the structure-directing agent was removed by calcination at 550°C for 5 h (heating rate: 1 K min^−1^).

The amino modification of the silica surface was performed via post-grafting (Fig 2B). Typically, 200 mg of NPSNPs were dispersed in 8 mL toluene and 40 μL APTES were added. The solution was stirred for 2 h at 80°C and 22 h at room temperature. Afterwards, the amino-modified nanoparticles (NH_2_-NPSNPs) were collected via centrifugation, washed three times with ethanol, and dried at 60°C.

### Characterization of NPSNPs

TEM images were taken on an FEI Tecnai G2 F20 TMP instrument (Hillsboro, USA). Size measurements were taken as the mean from 55 nanoparticles of each type with the software ImageJ 1.51j (Wayne Rasband, National Institutes of Health, USA). Each particle was measured twice. Dynamic light scattering (DLS) measurements were performed on a Zetasizer Nano ZSP from Malvern Instruments (Worcestershire, UK). For pH-dependent zeta potential titration curves, the Zetasizer Nano ZSP was used in combination with an MPT2 auto titrator from Malvern Instruments. Nitrogen physisorption analyses were performed at 77 K on a Quantachrome Autosorb-3 after outgassing the samples in vacuum at 80°C for 24 h. Surface areas were estimated by applying the Brunauer-Emmett-Teller (BET) equation. Pore size distributions were calculated using a non-linear density-functional theory (NLDFT) and fitting of the Quantachrome Kernel “N2 at 77 K on silica for cylinder pores, NLDFT equilibrium model” to the experimental data. Values for total pore volumes were determined at p/p_0 _= 0.92 to exclude interparticular volume. Thermogravimetric analysis (TGA) was measured with a Netzsch STA 429 CD analyzer, where samples were heated up to 1 000°C under an air atmosphere at a heating rate of 5 K min^−1^.

### Immobilization of VEGF and long-term release studies

VEGF immobilization and release studies were adapted from the previously reported work of our group [17]. The experiments were performed under sterile conditions in PBS supplemented with 0.1% BSA, which acted as a stabilizer for VEGF as well as filler protein to prevent protein adhesion to reaction tubes [18].

To sterilize the nanoparticles, samples were dispersed in ethanol (70%), centrifuged, and dried under a clean bench. Before use, these particles were exposed to UV radiation for 30 min. For immobilizing the growth factor, 4 mg nanoparticles (unmodified or amino-modified) were dispersed in 720 µL PBS (+0.1% BSA) and 80 µL VEGF solution (10 µg mL^−1^) were added. The resulting growth factor concentration was 1 µg mL^−1^ in a total volume of 800 µL. For an immobilization concentration of 2 µg mL^−1^, 4 mg NH_2_-NPSNPs were dispersed in 640 µL PBS (+0.1% BSA) and 160 µL of the VEGF solution were added. The incubation took place in a Thermomixer (Biozym Scientific, Hessisch Oldendorf, Germany) under constant shaking of 800 rpm at 4°C for 24 h. Afterwards, the nanoparticles were separated from the immobilization solution via centrifugation (10 min, 2 436 *g*), and the supernatant was removed. The particles were washed once with 800 µL fresh PBS (+0.1% BSA), followed by another centrifugation and supernatant removal step. All incubation and washing solutions were kept at −20°C until the VEGF content was quantified by ELISA. As a control, 4 mg of the respective nanoparticle type were treated under similar conditions but instead of the VEGF solution only PBS (+0.1% BSA) was added to nanoparticle dispersion.

Subsequent to immobilizing VEGF on the surface of NPSNPs, release experiments were performed. For this, fresh PBS (+0.1% BSA) was added to the particles, and samples were stored at 37°C. After certain time intervals, the particles were separated from the medium by centrifugation. The supernatant was carefully removed and replaced by fresh PBS (+0.1% BSA) before the release was continued at 37°C. Analogous to the incubation process all removed supernatants were stored at −20°C until VEGF quantification via ELISA.

### Immobilization of VEGF and subsequent release experiments for the tube assay

To simulate tube assay conditions, VEGF immobilization and release experiments with modified parameters (used particle amount and VEGF concentration) were performed. For preparing the incubation solution, 5 mg NH_2_-NPSNPs were sterilized as described before and dispersed in 2 mL PBS (+0.1% BSA). Subsequently, 80 µL of this dispersion (200 µg nanoparticles) were transferred to a micro reaction vessel (0.5 mL), 50 µL of VEGF solution (50 µg mL^−1^) and 70 µL of PBS (+0.1% BSA) were added, resulting in a total volume of 200 µL and a VEGF concentration of 12.5 µg mL^−1^. The following immobilization step and the release experiment were similar to those described before. The loading concentration was chosen based on the previous experiments with 1 µg mL^−1^ and 2 µg mL^−1^ VEGF incubation concentration. First, the amount of VEGF required for the tube formation was determined with dissolved VEGF. The applied loading concentration delivered a similar amount of VEGF within the duration of the tube assay. After incubation, the particles were first separated from the surrounding solution by centrifugation and then washed with 200 µL PBS (+0.1% BSA). The release started by adding 200 µL of fresh PBS (+0.1% BSA) and storage of samples at 37°C. All supernatants were kept at −20°C until VEGF quantification via ELISA. ELISA measurements were performed according to the manufacturer’s protocol.

### 2D sandwich tube assay

**Fig 3:**
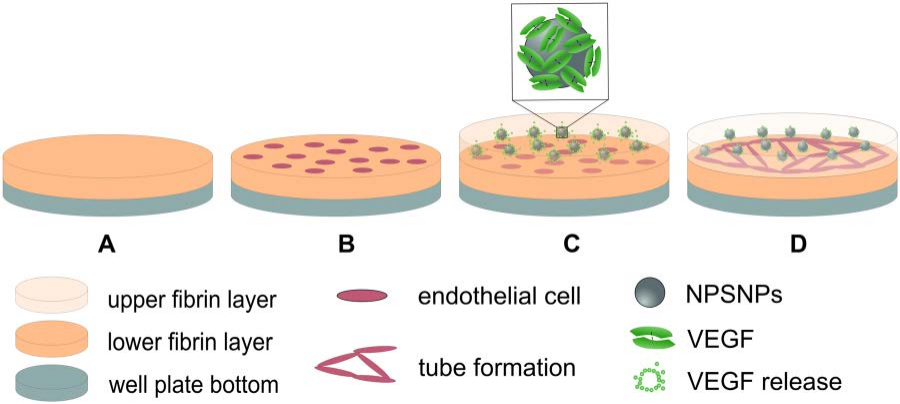
Schematic: set-up for the 2D sandwich tube assay. (A) bottom of the well plate covered with the lower layer of fibrin; (B) endothelial cells cultured on the surface of the lower fibrin layer; (C) addition of the upper fibrin layer with embedded NPSNPs releasing VEGF; (D) tube formation of endothelial cells after VEGF release.

To evaluate the influence of the developed VEGF release system on the tube formation of human umbilical vein endothelial cells (HUVECs) a two-dimensional fibrin-based tube assay was performed (Fig 3). In this assay, the cells were cultivated between two fibrin layers. The lower layer served as the substrate for cell cultivation, and the upper layer contained the VEGF-loaded nanoparticles enabling a release of the growth factor. For visualization of the vascular networks by fluorescence microscopy, red fluorescent protein-expressing HUVECs (RFP-HUVECs) were used. Cells were utilized in passages 5–7.

24 h before starting the tube assay, 200 µg NH_2_-NPSNPs were loaded with 12.5 µg VEGF as described. Washed particles were dispersed in 100 µL PBS with a pipette. Unloaded nanoparticles, treated under equal conditions, served as control. The prepared nanoparticle dispersions (c = 2 µg µL^−1^) were then incorporated into the second fibrin layer (see below).

The initial step of the assay was to cover wells of a 96-well plate with the first fibrin layer. For this, 1 µL of a thrombin solution and 50 µL of a fibrinogen solution (Table 1) were mixed to a final fibrin concentration of 5 mg mL^−1^ and allowed to polymerize for 30 min in each well. In the meantime, expanded endothelial cells were trypsinated and counted so that after polymerization 4 000 RFP-HUVECs in 200 µL M199 feeding medium could be added to the formed gel layer in each well. For cell attachment, the plate was placed in an incubator (37°C, 5% CO_2_) for 7 h. Components of the upper layer were premixed in another 96-well plate. On this occasion, 1 µL of thrombin solution was combined with 50 µL of a fibrinogen solution possibly plus further additive. The mixtures were transferred to the 96-well plate, on top of the first fibrin hydrogel layer (medium was previously removed). After another polymerization step (20 min), 200 µL feeding medium (M199 medium plus 40 ng mL^−1^ fibroblast growth factor (FGF), 50 µg mL^−1^ L-ascorbic acid-2-phosphate and 100 U mL^−1^ aprotinin (all from Sigma Aldrich, München, Steinheim, Germany) were added to each well. Finally, the plate was placed in an incubation oven (37°C, 5% CO_2_). Cells were cultivated for 7 days, changing the supernatant medium every 48 hours. One control was conducted without nanoparticles in the fibrin layer, but with additional 8 ng VEGF (concentration of 40 ng mL^−1^) in the medium yielding a total VEGF amount of 24 ng after two medium changes. This assay served as a positive control. As a negative control for the influence of VEGF on tube formation, VEGF was completely omitted in the feeding medium. For the evaluation of VEGF delivered from silica nanoparticles the assay was specified by resuspending 20 µg of VEGF-loaded NPSNPs in the upper gel, corresponding to a calculated release of 24 ng VEGF during the complete assay duration. Since NPSNPs per se could influence tube formation, further controls were performed: 20 µg unloaded NPSNPs were added to the upper gel as control, either with VEGF in the feeding medium or not. The groups tested in this assay are summarized in Table 2. Tube formation of each group was characterized by fluorescence microscopy (Nikon Eclipse TE300, Düsseldorf, Germany). The cells were monitored in regular intervals, showing the best results concerning tube formation after four days. Thus, at least three photographs were taken at this time point from each group. Tube formation was quantified via AngioTool software.

**Table 1:** Composition of fibrinogen and thrombin solution used for the two-dimensional tube assay (22 wells of a 96-well plate).

Solution type	Reagents	Volume/µL
Fibrinogen solution (in total 1 100 µL)	Medium (Gibco^TM^ M199)	929.72
	PBS	110.00
	Sodium hydroxide (1N)	3.30
	Fibrinogen	56.98
Thrombin solution (in total 22 µL)	Thrombin (0.275 U/100 µL stock)	6.05
	Calcium chloride (40 mM)	15.95

**Table 2:** Groups tested in the 2D sandwich tube formation assay to assess the suitability of VEGF-loaded NPSNPs as support for *in vitro* tube formation.

Group	VEGF	NPSNPs
A	3 x 8 ng (dissolved)	None
B	none	None
C	24 ng (calc. release)	VEGF-loaded
D	3 x 8 ng (dissolved)	Unloaded
E	None	Unloaded

VEGF: vascular endothelial growth factor; NPSNPs: nanoporous silica nanoparticles; calc: calculated.

## Results and discussion

### Characterization of the NPSNPs in comparison to NH_2_-NPSNPs

**Fig 4:**
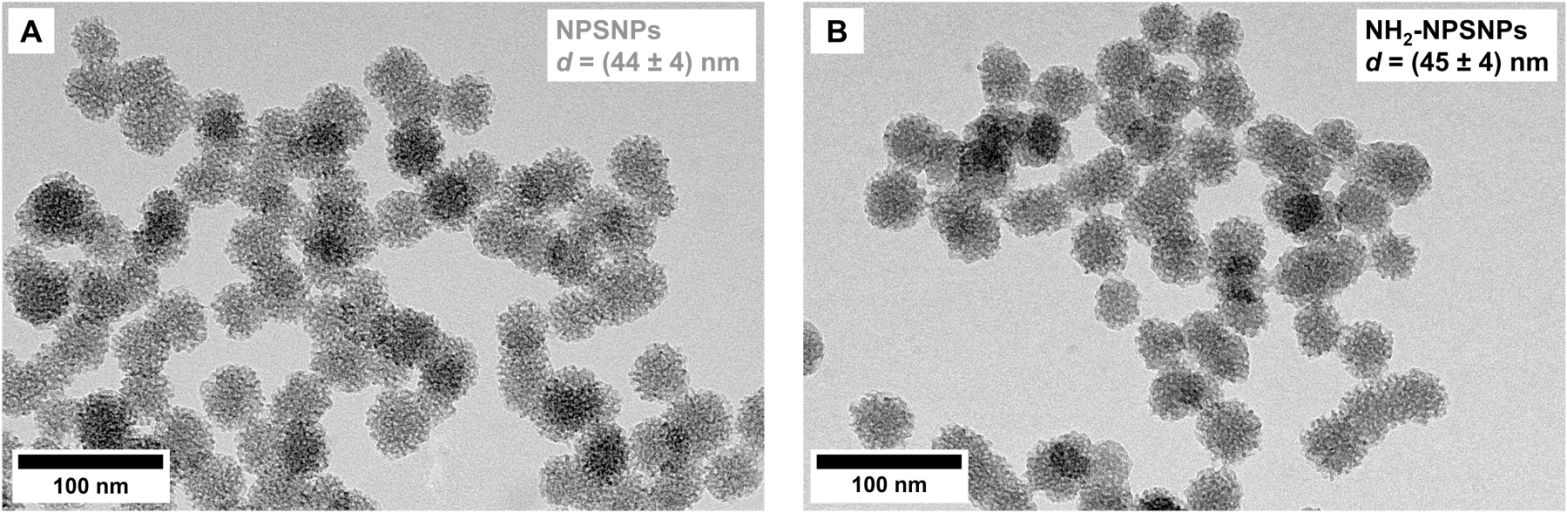
Characterization of NPSNPs: investigation of (A) unmodified NPSNPs in comparison to (B) amino-modified NPSNPs (NH_2_-NPSNPs) by transmission electron microscopy (d: diameter).

TEM and nitrogen physisorption measurements reveal the morphology and nanoporosity of the particles. TEM images demonstrate that the unmodified particles have a uniform size with a diameter of (44 ± 4) nm and are therefore lying in the nanometer range as depicted in Fig 4A. As additional information, the multiple spots of lighter color within the nanoparticles indicate the absence of material, thus a nanoporous system (Fig 4). As proof of the preservation of the particle morphology and their nanoporous system, the amino-modified nanoporous silica nanoparticles were characterized by TEM as well. After amino modification, the nanopores are still visible in TEM images (Fig 4B) and the nanoparticles have a similar diameter of (45 ± 4) nm.

**Fig 5:**
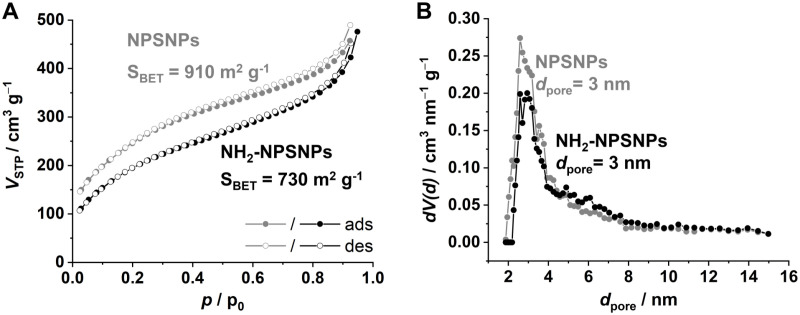
Characterization of nanopores by nitrogen physisorption: (A) nitrogen physisorption isotherms at 77 K of unmodified (grey) and amino-modified (NH_2_-NPSNPs, black) NPSNPs (ads: adsorption branch; des: desorption branch); (B) pore size distribution from unmodified (grey) and NH_2_-NPSNPs (black) calculated by NLDFT.

The presence of nanopores can also be drawn from the nitrogen physisorption measurement shown in Fig 5. The isotherms (recorded at 77 K) of the unmodified and the amino-modified NPSNPs exhibit a typical type IV hysteresis of a mesoporous, or as it can also be named, nanoporous material [24,25]. For the unmodified NPSNPs, the evaluation with the BET model [26] [ISO9277:2014-01] leads to an inner surface area of 910 m^2^ g^−1^ and NLDFT [27] [ISO-15901–2:2022] analysis to a pore size of 3 nm. The total pore volume up to the shown relative pressure was determined to a value of 0.71 cm^3^ g^−1^. Further nitrogen physisorption measurements exhibit a decrease of the inner surface area to 730 m^2^ g^−1^ due to the filling of the pore volume by the amino groups (Fig 5A). Another possible effect is the pore blocking by amino groups situated at the pore openings. The pore diameter stays unaffected with a value of 3 nm calculated by NLDFT (Fig 5B). The total pore volume is slightly reduced to 0.65 cm^3^ g^−1^.

**Fig 6:**
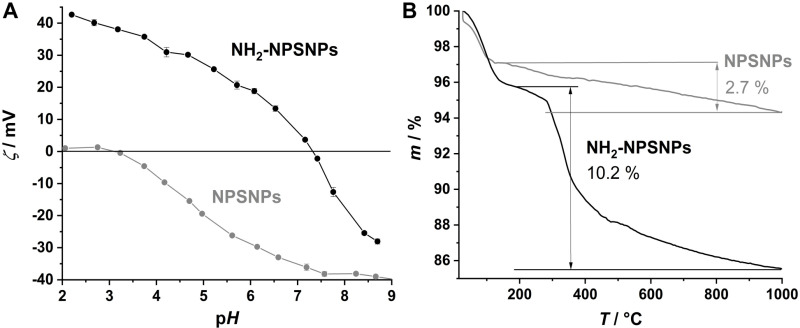
Characterization of unmodified NPSNPs in comparison to amino-modified NPSNPs (NH_2_-NPSNPs): (A) pH-dependent zeta-potential measurements of unmodified (grey) and NH_2_-NPSNPs (black); (B) thermogravimetric measurements of unmodified NPSNPs (grey) and NH_2_-NPSNPs (black). The mass loss of the second step results in an amino group density of 430 µmol g^−1^.

The unmodified NPSNPs possess a zeta potential close to 0 mV in the pH range from 2 to 3 (Fig 6A). At pH of 3.1 the isoelectric point occurs. From that point on the zeta potential is decreasing to a value of about −39 mV at pH 8.7. This increasing negative charge of the silica nanoparticles is caused by the progressing deprotonation of the weakly acidic silanol groups (-Si-OH → -Si-O^−^) and is typical for nanoporous silica materials [28]. The zeta potential of the NH_2_-NPSNPs abates over the whole pH range from 2 to 9 (Fig 6A). The difference is about 70 mV. At pH 2 the potential is with 43 mV much more positive than for the unmodified NPSNPs. The zeta potential stays positive up to the isoelectric point at pH 7.3 and then decreases to a value of −28 mV at pH 8.7. These positive potentials are proof of a successful amino modification. In the acidic pH range the amino groups are protonated (-N^+^H_3_) and therefore positively charged, with ongoing deprotonation the amino groups become neutral at pH 7.3 and are then even negatively charged (-N^−^H) at lower pH values [17]. The successful amino modification is confirmed by thermogravimetric measurements as well. After the mass loss up to the range of 200°C which can be ascribed to the loss of adsorbed water the NH_2_-NPSNPs exhibit a higher mass loss of 10.3% in comparison to 2.7% in the case of the unmodified ones (Fig 6B).

This difference is evoked by the combustion of the organic moieties of the aminopropyl residues attached to the silica surface, whereas the mass of the unmodified silica is only reduced by the further condensation reaction of the silanol groups (-Si-OH^…^HO-Si-) to siloxane (-Si-O-Si-) bridges. The amino group density calculated from this difference gives an estimated value of 430 µmol g^−1^. With the typical amount of three to four silanol groups per nm^2^ of the surface of calcined nanoporous silica [29,30] and a BET surface area of 910 m^2^ g^−1^ for the unmodified NPSNPs, a range from 4.5 mmol g^−1^ to 6 mmol g^−1^ present silanol groups can be calculated. With the given amino group density this leads to a range of 7% to 9% amino-modified silanol groups. For comparison, we also carried out a calorimetric assay using the negatively charged dye Orange II. We quantified the amount of dye molecules that were bound to the positively charged amino groups by UV/vis spectroscopy (further information given in the ESI). This led to an amino group density of 36 µmol g^−1^ and thus 0.6% to 0.8% modified silanol groups which is lower than calculated from thermogravimetric measurements. Since the thermogravimetric measurements are less accurate due the high residual mass of the NPSNP and the resulting low percentage mass loss and since it is not possible to accurately distinguish between silica condensation and organic combustion, we consider the calorimetric determination to be more reliable. Overall, the amount of amino-modified silanol groups is sufficient to achieve the desired surface properties as proven by zeta potential measurements.

### Aggregation behavior of VEGF

**Fig 7:**
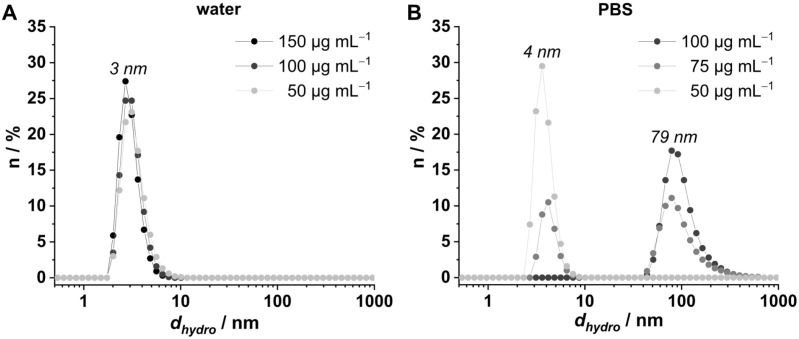
Aggregation behavior of VEGF: dynamic light scattering measurements of VEGF at different concentrations in (A) water and (B) phosphate-buffered saline (PBS). (d_hydro_: hydrodynamic diameter).

The aggregation behavior of the VEGF was investigated in water (Fig 7A) and PBS (Fig 7B) at different concentrations by dynamic light scattering. In water, only one maximum for the hydrodynamic diameter of 3 nm for all concentrations can be found (50 µg mL^−1^, 100 µg mL^−1^, and 150 µg mL^−1^). This particle diameter lies in the same range as the hydrodynamic diameter of 5.2 nm assumed by Erickson’s formula and of 6.3 nm calculated from crystal structure for the dimeric VEGF protein [31].

The variation can be evoked by estimations within the theoretical calculations or different experimental conditions. Additionally, the mismatch can be evoked by the assumption of a spherical shape for DLS calculations, which is not valid for VEGF, that is present in an elongated form. In PBS a concentration-dependent aggregation behavior can be recognized. At the lowest concentration of 50 µg mL^−1^ a single species with a diameter of 4 nm occurs. By increasing the concentration to 75 µg mL^−1^ a second maximum at 79 nm due to protein aggregation is present. This diameter becomes the only particle diameter at a concentration of 100 µg mL^−1^. Based on these results VEGF concentrations lower than 50 µg mL^−1^ were applied in this study.

### VEGF release

**Fig 8:**
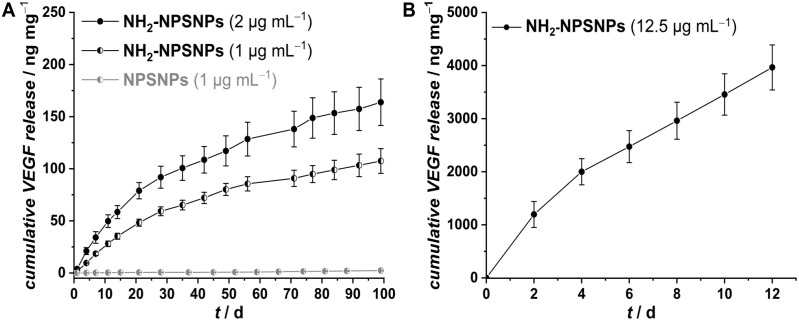
Cumulative VEGF release from NPSNPs loaded in differently concentrated VEGF solutions: (A) unmodified NPSNPs (grey/white) and amino-modified (NH_2_-NPSNPs, white/black) NPSNPs loaded in VEGF solution with a concentration of 1 µg mL^−1^, and 2 µg mL^−1^ (black) for NH_2_-NPSNPs; (B) NH_2_-NPSNPs loaded in a VEGF solution with a concentration of 12.5 µg mL^−1^.

After loading of VEGF onto the nanoparticles the release behavior was investigated. For the unmodified NPSNPs incubated in a VEGF solution of 1 µg mL^−1^ nearly no released VEGF, (2.4 ± 0.6) ng mL^−1^ after 99 days in PBS (+0.1% BSA) at 37°C can be detected (Fig 8A). In comparison, the NH_2_-NPSNPs show a continuous release of up to (107.5 ± 11.9) ng mg^−1^ of the nanoparticles over the same period. By using a higher VEGF incubation concentration of 2 µg mL^−1^ the release can be increased up to (163.8 ± 22.4) ng mg^−1^ (Fig 8A). Assuming that all of the offered VEGF was loaded on the NPSNPs (200 ng mg^-1^ for 1 µg mL^−1^; 400 ng mg^−1^ for 2 µg mL^−1^), 54% in case of the 1 µg mL^−1^ incubation concentration and 41% in case of 2 µg mL^−1^ were released. In another study with the protein TGF-β3 (transforming growth factor β3) bound to silica nanoparticles similar amounts of released protein were achieved (115 ng mg^−1^ TGF-β3 after 77d, 95 ng mg^−1^ VEGF after 77d) [17]. Fitting the release profiles with zero order and first order kinetic model showed better fit to first order kinetics and thus a concentration dependent behavior (S2 Table in S1 File). This can be explained by the higher VEGF concentration on the nanoparticle surface in contrast to the medium. Furthermore, fitting with Higuchi model, which predicts dependence of the released amount and the square root of time, revealed a diffusion-controlled release. As the VEGF is too large to fit into the pores of the NPSNPs more complex kinetic models were not considered [32]. Based on these data, we conducted another release experiment with a higher VEGF concentration of 12.5 µg mL^−1^ over 12 d analog to the 2D sandwich tube assay to determine the mass of VEGF-loaded NPSNPs needed to deliver the same amount of VEGF as given in dissolved form within the tube assay (Fig 8B). Here, a much higher release could be achieved, within 12 d an amount of nearly 4 µg VEGF per mg nanoparticles was released which corresponds to 32% of the offered VEGF amount. The higher release is due to higher loading and the resulting higher concentration gradient between the surface and the medium. Additionally, the lower nanoparticle concentration and more frequent medium changes performed, which were carried out according to the tube assay accelerated the release. Fitting with a kinetic model also resulted in a first order, diffusion-controlled release (S2 Table in S1 File). In a study by Kim *et al.* similar effects of VEGF release from VEGF-loaded NPSNPs incorporated into a porous collagen scaffold were seen. In this study, 140 ng per mg NPSNPs were released over 28 d which corresponds to 56% of the total incorporated amount. As the reason for the incomplete release, the strong interaction of the growth factor with the positively charged nanoporous silica surface is given [33]. Further studies will require extended characterization, e.g., by fluorescence spectroscopy, of the VEGF released at later time points, since the ELISA can only detect its immunological activity.

### 2D sandwich tube assay

The 2D sandwich tube assay was applied to evaluate the impact of VEGF release from NPSNPs on tube formation capabilities of endothelial cells (ECs). Therefore, RFP-HUVECs were cultured between two layers of a fibrin gel under the influence of differently delivered VEGF. The aim was to compare the effect of VEGF that was dissolved in medium and given to the cells with VEGF gradually released from NPSNPs to achieve an autonomous delivery system for implantable tissue constructs.

**Fig 9:**
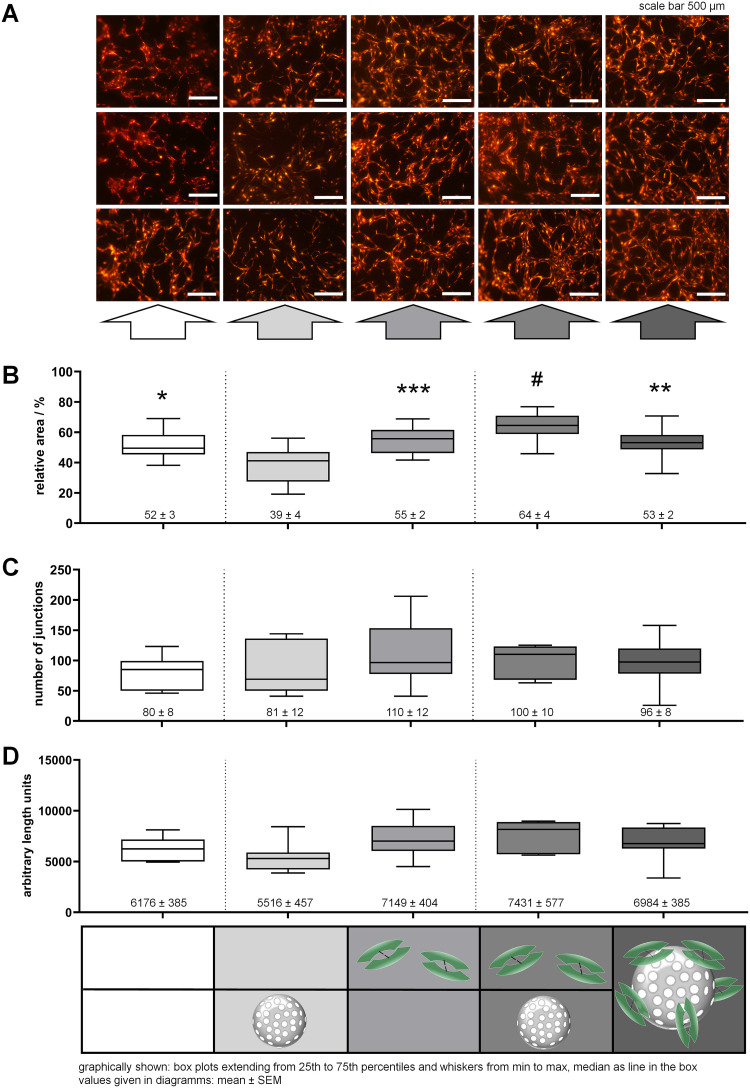
Dissolved VEGF and VEGF delivered by NPSNPs achieve equal tube formation. (A) Microscopic images of the tube formation of RFP-HUVECs within the 2D sandwich assay: images were obtained 4 days after the start of the experiment. Wells supplied with (white) neither dissolved VEGF nor NPSNPs, (light grey) only unloaded NPSNPs, (middle grey) dissolved VEGF, (grey) dissolved VEGF and unloaded NPSNPs, and (dark grey) VEGF loaded on NPSNPs were analyzed. All NPSNPs were amino-modified. Best tube formation was achieved in wells receiving only dissolved VEGF (middle grey) and wells receiving VEGF delivered by NPSNPs (dark grey). Poor tube formation was seen in wells receiving neither VEGF nor NPSNPs (white) and wells receiving unloaded NPSNPs with VEGF in medium (grey). No tube formation was seen in wells not receiving VEGF but unloaded NPSNPs (light grey). Scale bar: 500 µm. (B) Quantification of the tube formation of RFP-HUVECs within the 2D sandwich assay: AngioTool quantifications concerning (B) relative tube area, (C) number of junctions, and (D) total vessel length. The bars represent the values for cells treated with (white) neither dissolved VEGF nor NPSNPs, (light grey) only unloaded NPSNPs, (middle grey) dissolved VEGF, (grey) dissolved VEGF and unloaded NPSNPs, and (dark grey) VEGF loaded on NPSNPs. Graphically shown: box plots extending from 25th to 75th percentiles and whiskers from min to max, median as line in the box, values given in diagrams: mean ± SEM, RFP-HUVECs: red-fluorescent protein-expressing human umbilical vein endothelial cells, VEGF: vascular endothelial growth factor, NPSNPs: nanoporous silica nanoparticles. *: p < 0.05; **: p < 0.01; ***: p < 0.001; #: p < 0.000. Significances shown against only unloaded NPSNPs.

The tube formation of RFP-HUVECs in each well of the 2D sandwich assay was investigated on day four of the assay by fluorescence light microscopy. Wells of the positive control, which received repeated doses of VEGF with every renewal of medium showed the highest level of tube formation (Fig 9A, 3^rd^ column, middle grey). Closest to that, the wells that obtained 20 µg VEGF-loaded NPSNPs (Fig 9A, 5^th^ column, dark grey) generated comparable tube formation by RFP-HUVEC as seen in wells of the positive control. The interconnectivity of the freshly formed tubes was high in both groups, but in the case of the VEGF-loaded NPSNPs, they appear to be more evenly distributed throughout the wells. When unloaded NPSNPs were given to the cells and dissolved VEGF was added (Fig 9A, 4^th^ column, grey), tube formation was lower than in the two before-mentioned groups. Additionally, the cells here are arranged rather into broader patches than into an interconnected tube network. In the two groups that did not receive any VEGF (Fig 9A, 1^st^ and 2^nd^ column, white and light grey), poor to no vessel formation was observed. In wells where the cells were neither supplied with NPSNPs nor VEGF (Fig 9A, 1^st^ column, white) only minimal tube formation could be recognized, although cells mainly grew into patches evenly covering the gel area. Cells that were provided with unloaded NPSNPs but no VEGF showed the poorest tube formation (Fig 9A, 2^nd^ column, light grey). Here, cells only partially grew into patches and hardly formed any network structures. In some wells ECs seemed to elongate in a try to interconnect, however, no real tube formation could be observed (Fig 9A, 2^nd^ column, bottom image). Thus, VEGF released from NPSNPs exerted the same biological function as dissolved VEGF added freshly and repeatedly to the cells.

For a better quantification of the 2D sandwich assay, the experiment was evaluated with the software AngioTool [34], analyzing the relative tube area, number of junctions, and the total vessel length (Fig 9B–D). Overall, it can be stated that the group with added unloaded NPSNPs and no VEGF shows the lowest values (Fig 9, light grey). The presence of the silica, and consequently silicon ions seem to disturb the cells if no VEGF is present to support their tube formation. If nothing than medium is supplemented to the cells slightly higher values can be reached (Fig 9, white). VEGF-loaded NPSNPs achieve similar results compared to when VEGF is added in dissolved form or dissolved VEGF is given in combination with non-loaded NPSNPs. Concerning the relative tube area, no significant difference between the control (49%, no supplementation) and the three samples with VEGF present (55% dissolved VEGF, 64% dissolved VEGF + unloaded NPSNPs, 53% VEGF-loaded NPSNPs) was found, but all exhibit a significantly higher relative tube area compared to the group of only added unloaded NPSNPs. In the case of the number of junctions and the total vessel length, no significant differences can be found, but the tendency of a similar level for the three VEGF-containing groups can also be observed here. Similar effects could also be observed by Kim *et al*. They found a significantly increased number of newly formed blood vessel complexes in a chick chorioallantonic membrane (a highly vascularized membrane present in eggs) model when applying a collagen scaffold with incorporated VEGF-loaded NPSNPs whereas the total vessel length, size, and total junctions showed only a positive tendency for VEGF-loaded samples [33].

### Effect of sustained VEGF delivery on the tube formation of ECs

In the presented study the sustained delivery of VEGF from NPSNPs in comparison to repeated fresh doses of VEGF in an endothelial tube formation assay between two layers of fibrin hydrogel was investigated. NPSNPs were used as drug delivery vehicles to constantly deliver the same total amount of VEGF (24 ng) to ECs in a tube formation assay, as given gradually and freshly with every media change. Due to the short half-life of exogenous growth factors, repeated delivery of fresh VEGF to the tissue construct would be needed for long-term *in vitro* or *in vivo* applications. This procedure is rather impractical *in vitro* and nearly impossible *in vivo* [35]. To overcome this major issue a drug delivery system of VEGF-loaded NPSNPs was applied. The objective of this study was to check whether VEGF bound to and released from NPSNPs would exert the same biological effect as freshly added VEGF. The results of the 2D sandwich tube assay showed clear evidence that ECs in fibrin gels receiving sustained VEGF delivered by NPSNPs showed similar if not even better, tube formation compared to the control cells receiving fresh VEGF doses with every medium change (Fig 9). Consequently, the attachment of VEGF to NPSNPs did not evoke any conformational changes interfering with its biological action. The effect of pure NPSNPs without loaded VEGF on ECs depended strongly on the presence or absence of VEGF. While cells treated with unloaded NPSNPs and freshly added VEGF in the media exhibited some tube formation, although formed networks appeared rather patchy, cells treated with NPSNPs and receiving no VEGF demonstrated no tube formation capacity. This leads to the assumption that NPSNPs alone do not elicit a positive effect on endothelial tube formation in a monoculture of ECs, and VEGF needs to be supplemented in one form or the other. This finding stands in contrast to other studies where silica itself was described as having pro-angiogenic properties [36–38], but in these studies, the silica was directly given to the cells. In the present study, the cells were spatially separated from the silica source due to the incorporation in the fibrin gel. Jo *et al.* on the other hand have found in their study that too high concentrations of released Si^4+^ might also have inhibitory effects on endothelial tube formation [39]. The need for VEGF in tube formation assays was also described in a study by Feng *et al.* [40].

The present assay was performed in the absence of supporting cell types, such as adipose-derived stem cells (ASCs), as these are known to produce VEGF themselves. Monocultures of ECs have proven to rather proliferate than form tubes and enlarge newly formed capillaries [41,42]. This effect can also be seen in our data provided elsewhere [43]. Here, ECs were grown in co-culture with ASC and formed vessel networks that appeared to feature narrower and interconnected capillary formation. A study by Rohringer *et al.* has also suggested that ECs need direct cell-cell contact with ASCs rather than soluble VEGF alone to achieve proper tube formation [44]. However, a very attractive advantage of the integration of VEGF-loaded NPSNPs is the autonomous and constant release of the growth factor, albeit cellular growth factor secretion can never be controlled evenly in total. However, with this specially developed 2D sandwich tube assay applying only ECs the possible maintenance of the biological activity of VEGF delivered from NPSNPs even in the absence of tube formation supporting ASCs was demonstrated. Their tube formation was enabled and even positively influenced.

Moreover, for future studies, the next development to be evaluated will be, whether better vessel formation will be achieved when not only supplying VEGF in a sustained manner via NPSNPs but also other growth factors, such as platelet-derived growth factor (PDGF), which are rather involved in vessel maturation than in sprouting, to produce a more stable network over a longer period [45]. Also, the addition of growth factor-loaded NPSNPs in a co-culture of ECs with supporting cell types such as ASCs is of interest.

## Conclusions

With this study, we demonstrated that long-term VEGF delivery is possible with the application of NPSNPs as a drug carrier. The loading of appropriate amounts of VEGF onto the NPSNPs was achieved by amino modification via a grafting approach and could be adjusted by the variation of the VEGF loading concentration. The VEGF showed to be stable in a release experiment in PBS *in vitro* over the whole release of 100 days as proved by ELISA determination. In a specially developed 2D sandwich assay the VEGF-loaded NPSNPs led to a similar extent of tube formation of ECs in comparison to repeatedly added doses of VEGF which makes them highly suitable for use in constructs for vascular tissue engineering.

## Supporting information

S1 File Additional data on literature and analytical methods. S1 Table. Examples of VEGF material-based release systems in the literature. S1 Fig. Calibration curve for the Orange II assay for amino group density quantification (and further details). S2 Table. Values for the calculated coefficient of determination R^2^ for the different kinetic models (and further details).
(PDF)

S2 File Raw data set. (XLSX)
