# Supplementary material for: Sustained delivery of functional vascular endothelial growth factor from nanoporous silica nanoparticles into a fibrin gel
Source: PLoS One. 2025 Jun 26;20(6):e0326561. doi: 10.1371/journal.pone.0326561 (PMC12200659; doi:10.1371/journal.pone.0326561)
Supplement: S1 File — S1 Table. Examples of VEGF material-based release systems in the literature. S1 Fig. Calibration curve for the Orange II assay for amino group density quantification (and further details). S2 Table. Values for the calculated coefficient of determination R2 for the different kinetic models (and further details). (PDF) [file pone.0326561.s001.pdf]

Electronic supplementary information (ESI)

Sustained delivery of functional vascular endothelial growth factor from nanoporous silica nanoparticles into a fibrin gel

Karen Besecke[‡a,d], Sarah Zippusch[b,d], Florian Helms[b,d], Ulrike Böer[b,d], Mathias Wilhelmi[c,d], Peter Behrens[a,d], and Nina Ehlert[*a,d]

[a]Institute of Inorganic Chemistry, Leibniz University Hannover, Callinstraße 9, 30167 Hannover, Germany.
[b]Department of Cardiothoracic-, Transplantation, and Vascular Surgery, Hannover Medical School, Carl-Neuberg-Straße 1, 30625 Hannover, Germany.
[c]Department of Vascular- and Endovascular Surgery, St. Bernward Hospital, Treibestraße 9, 31134 Hildesheim, Germany.
[d]NIFE – Lower Saxony Center for Biomedical Engineering, Implant Research and Development, Stadtfelddamm 34, 30625 Hannover, Germany.
[‡]Cardior Pharmaceuticals GmbH, Holleritallee 20, 30419 Hannover, Germany

S1 File. Additional data on literature and analytical methods

S1 Table. Examples of VEGF material-based release systems in the literature.

drug release system for VEGF	lit
**particle-based systems**	
VEGF immobilized on silica microparticles (≈250 µm)	1
VEGF/RunX2-siRNA loaded on nanoporous silica nanoparticles (50 nm)	2
VEGF loaded on silica supraparticles	3
VEGF loaded into nanoporous hydroxyapatite	4
VEGF encapsulated in alginate microparticles (10 µm)	5
VEGF encapsulated in PLGA particles (≈400 nm; 70-80 µm; 4-10 µm; ≈5 µm; 200 nm)	6–10
VEGF encapsulated in peptide cross-linked acrylamide nanocapsules	11
**matrix-based systems**	
VEGF encapsulated in alginate gel (10 µm)	12
VEGF incorporated in alginate/chitosan/PLA scaffolds	13
VEGF incorporated into collagen hydrogels	14
VEGF/PDGF immobilized on a PLGA matrix	15
VEGF incorporated into PLLA fiber mats	16
VEGF loaded into cross-linked gelatin fiber mats	17
VEGF immobilized on desulfated heparin derivatives-star-shaped PEG hydrogels	18
VEGF entrapped in gelatin-coated porous PCL scaffolds	19
VEGF incorporated into PEG maleimide hydrogel	20
VEGF immobilized in PEG/cross-linked matrix metalloproteinase scaffolds	21
VEGF incorporated in to PEG-(PCL/PEG) core-shell nanofibers	22
VEGF incorporated into collagen-coated PCL scaffolds	23
VEGF immobilized on heparin-coated PCL/keratin fiber mats	24
VEGF immobilized on heparin-coated gelatin/PCL fiber scaffolds	25
VEGF loaded into photo-cross-linked trimethylene carbonate/PCL/PLA	26
**combination of particle & matrix**	
VEGF immobilized on silica nanoparticles (≈200 nm) embedded within a collagen matrix	27
VEGF encapsulated in PLGA microparticles (77 µm) in combination with fibrin glue	28
VEGF encapsulated in PLGA microparticles (21 µm) in combination included in collagen scaffolds	29
VEGF encapsulated in PLGA nanoparticles (112 nm) on PVA fiber mats	30
VEGF immobilized on heparin-functionalized PLGA nanoparticles (160 nm), embedded in fibrin	31,32
VEGF/MPC-1 encapsulated in alginate microparticles (9 µm), embedded in a collagen/fibronectin gel	33
VEGF loaded into heparin/chitosan nanoparticle-immobilized decellularized bovine jugular vein scaffold	34
VEGF encapsulated in dextran sulfate/chitosan nanoparticles (≈460 nm) incorporated in Matrigel™ & PLGA scaffolds	35

MCP-1: monocyte chemotactic protein 1; PDGF: platelet-derived growth factor; PLA: poly(lactide); PLGA: poly(lactide-*co*-glycolide; PLLA: poly(L-lactic acid) PEG: polyethylene glycol, PCL: poly(e-caprolactone); PVA: poly(vinyl alcohol)

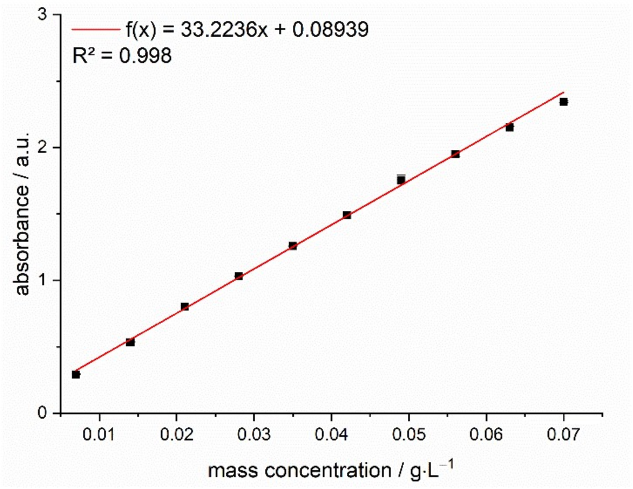

S1 Fig. Calibration curve for the Orange II assay for amino group density quantification.

Further details on amino group density quantification assay with Orange II

Chemicals

Tropaeolin 000 Nr. 2 (Orange II, ≥85%) and hydrochloric acid (37%) were purchased from Sigma-Aldrich Corporation (München, Germany).

Orange II assay

The determination of amino groups on the particle surface was conducted using modified version of the Orange II assay [36]. All experiments were carried out in triplicate. In brief, 10 mg of (amino-modified) NPSNPs were dispersed for 10 seconds in 1.5 mL of a 0.7 $g \cdot L^{-1}$ concentrated acidic Orange II solution using a vortex mixer. The acidic solution was prepared by dissolving 3.5 g of Orange II in 500 mL of water with the addition of 0.1 mL of hydrochloric acid, resulting in a pH value of 3. Subsequently, the particles were incubated at 37 °C in a convection oven to allow diffusion of the negatively charged Orange II dye to the positively charged amino groups. Because it was not possible to desorb the dye molecule completely from the surface of the NPSNPs, the Orange II concentration was determined before and after the incubation to calculate the number of attached Orange II molecules. After 30 minutes, the particles were separated by centrifugation for 10 minutes at 10,000 rpm. Following centrifugation, 200 µL of each amino modified sample supernatant was removed three times and analyzed by UV/Vis spectroscopy at 484 nm. A calibration curve was established over a range of 0.007 $g \cdot L^{-1}$ to 0.07 $g \cdot L^{-1}$ (S1 Fig). The measurements were performed on a Spark 10 M (Tecan Trading AG, Männedorf, Switzerland). As comparative sample unmodified NPSNP were tested. The procedure for these was the same as for the amino-modified particles. Because the absorbance of the supernatants was too high to measure in case of the unmodified NPSNP, 100 µL of the supernatant were taken and diluted with 900 µL of hydrochloric acid solution. At the end, the calculated number of amino-groups on the unmodified particles (3.2 (±0.3) µmol amino groups per gram) was subtracted from the calculated number of amino-groups on the amino-modified particles (39.3 (±1.1) µmol amino groups per gram) to exclude unspecific adsorption. This gave a value of 36 (±1.2) µmol amino groups per gram particles, with an inner surface of 730 m^2 per gram, the density of amino groups in one gram particle is 0.049 $\mu mol \cdot m^{-2}$ (or $2.9 \cdot 10^{16}$ amino groups per m^2). A typical amount of silanol groups of calcined nanoporous silica is two to four silianol groups per nm^2, thus $2 \cdot 10^{18}$ to $4 \cdot 10^{18}$ silanol groups per m^2. Taking this into account, about 0.7% to 1.5% of the present silanol groups were modified.

S2 Table. Values for calculated coefficient of determination R^2 for the different kinetic models.

incubation concentration /µg mL^{-1}	R^2		
	zero order	first order	Higuchi
1	0.919	0.963	0.989
2	0.919	0.953	0.993
12.5	0.965	0.982	0.986

Further details on release kinetics of VEGF release

Recorded release profiles with the incubation concentration 1 µg mL^{-1}, 2 µg mL^{-1} and 12.5 µg mL^{-1} were fitted with different kinetic models.

1. Zero order kinetic model: $\frac{M_t}{M_0} = K_0 t$
2. First order kinetic model: $logQ_t = logQ_0 - \frac{K_1}{2.303}$
3. Higuchi model: $Q = K_H \sqrt{t}$

M_t is the fraction of the drug released at time t, M_0 is the initial drug fraction, K_0 is the zero order release constant, K_1 is the first order release constant, K_H is the Higuchi constant, t is the time, Q_t is the amount of drug released in time t , Q is the drug amount released in time t per unit area

References

1 K. Dashnyam, G. Z. Jin, J. H. Kim, R. Perez, J. H. Jang and H. W. Kim, *Biomaterials*, 2017, 116, 145–157.

2 W. Yu, X. Zhu, J. Liu, and J. Zhou, *Bioengineering*, 2023, 10, 859–878.

3 K. H. Gurner, J. J. Richardson, A. J. Harvey and D. K. Gardner, *Human Reproduction*, 2021, **36**, 1776–1783.

4 C. K. Poh, S. Ng, T. Y. Lim, H. C. Tan, J. Loo and W. Wang, *J Biomed Mater Res A*, 2012, **100 A**, 3143–3150.

5 S. M. Jay and W. M. Saltzman, *J Control Release*, 2009, **134**, 26–34.

6 J. S. Golub, Y. T. Kim, C. L. Duvall, R. V. Bellamkonda, D. Gupta, A. S. Lin, D. Weiss, W. R. Taylor and R. E. Guldberg, *Am J Physiol Heart Circ Physiol*, 2010, **298**, 1959–1965.

7 B. Zhao, X. Y. Liu, H. J. Ding, L. Zhong, Y. Sun, R. Hong, Y. Y. Qu, J. J. Wang, X. P. Yang, M. Lu, H. T. Sun and X. H. Li, *J Clin Neurosci*, 2020, **81**, 122–132.

8 F. R. Formiga, B. Pelacho, E. Garbayo, G. Abizanda, J. J. Gavira, T. Simon-Yarza, M. Mazo, E. Tamayo, C. Jauquicoa, C. Ortiz-de-Solorzano, F. Prósper and M. J. Blanco-Prieto, *J Control Release*, 2010, **147**, 30–37.

9 E. Herrán, R. Pérez-González, M. Igartua, J. L. Pedraz, E. Carro and R. M. Hernández, *J Control Release*, 2013, **170**, 111–119.

10 O. Qutachi, A. J. Bullock, G. Gigliobianco and S. MacNeil, *Int J Pharm*, 2019, **561**, 236–243.

11 S. Zhu and T. Segura, *Ann Biomed Eng*, 2016, **44**, 1983–1992.

12 E. A. Silva and D. J. Mooney, *Biomaterials*, 2010, **31**, 1235–1241.

13 B. De la Riva, C. Nowak, E. Sánchez, A. Hernández, M. Schulz-Siegmund, M. K. Pec, A. Delgado and C. Évora, *Eur J Pharm Biopharm*, 2009, **73**, 50–58.

14 Y. Tabata, M. Miyao, M. Ozeki and Y. Ikada, *J Biomater Sci Polym Ed*, 2000, **11**, 915–930.

15 T. P. Richardson, M. C. Peters, A. B. Ennett and D. J. Mooney, *Nat Biotechnol*, 2001, **19**, 1029–1034.

16 H. J. Chung, J. T. Kim, H. J. Kim, H. W. Kyung, P. Katila, J. H. Lee, T. H. Yang, Y. Il Yang and S. J. Lee, *J Control Release*, 2015, **205**, 218–230.

17 C. Del Gaudio, S. Baiguera, M. Boieri, B. Mazzanti, D. Ribatti, A. Bianco and P. Macchiarini, *Biomaterials*, 2013, **34**, 7754–7765.

18 U. Freudenberg, A. Zieris, K. Chwalek, M. V. Tsurkan, M. F. Maitz, P. Atallah, K. R. Levental, S. A. Eming and C. Werner, *J Control Release*, 2015, **220**, 79–88.

19 W. L. Xu, H. S. Ong, Y. Zhu, S. W. Liu, L. M. Liu, K. H. Zhou, Z. Q. Xu, J. Gao, Y. Zhang, J. H. Ye and W. J. Yang, *Tissue Eng Part A*, 2017, **23**, 445–457.

20 E. A. Phelps, K. L. Templeman, P. M. Thulé and A. J. García, *Drug Deliv Transl Res*, 2015, **5**, 125–136.

21 A. H. Zisch, M. P. Lutolf, M. Ehrbar, G. P. Raeber, S. C. Rizzi, N. Davies, H. Schmökel, D. Bezuidenhout, V. Djonov, P. Zilla and J. A. Hubbell, *The FASEB Journal*, 2003, **17**, 2260–2262.

22 H. Zigdon-Giladi, A. Khutaba, R. Elimelech, E. E. Machtei and S. Srouji, *J Biomed Mater Res A*, 2017, **105**, 2712–2721.

23 S. Singh, B. M. Wu and J. C. Y. Dunn, *J Biomed Mater Res A*, 2012, **100 A**, 720–727.

24 X. Wan, P. Li, X. Jin, F. Su, J. Shen and J. Yuan, *J Biomed Mater Res A*, 2020, **108**, 292–300.

25 K. Wang, X. Chen, Y. Pan, Y. Cui, X. Zhou, D. Kong and Q. Zhao, *Biomed Res Int*, 2015, ID 865076, DOI:10.1155/2015/865076.

26 R. Chapanian, M. Y. Tse, S. C. Pang and B. G. Amsden, *J Pharm Sci*, 2012, **101**, 588–97.

27 J. H. Kim, T. H. Kim, M. S. Kang and H. W. Kim, *Biomed Res Int*, 2016, ID 9676934, DOI:10.1155/2016/9676934.

28 L. Zhang, L. Zhang, X. Lan, M. Xu, Z. Mao, H. Lv, Q. Yao and P. Tang, *J Mater Sci Mater Med*, 2014, **25**, 1165–1172.

29 C. Borselli, F. Ungaro, O. Oliviero, I. D'Angelo, F. Quaglia, M. I. La Rotonda and P. A. Netti, *J Biomed Mater Res A*, 2010, **92**, 94–102.

30 K. Nagase, Y. Nagumo, M. Kim, H. J. Kim, H. W. Kyung, H. J. Chung, H. Sekine, T. Shimizu, H. Kanazawa, T. Okano, S. J. Lee and M. Yamato, *Macromol Biosci*, 2017, 1700073, DOI:10.1002/mabi.201700073.

31 Y. Il Chung, S. K. Kim, Y. K. Lee, S. J. Park, K. O. Cho, S. H. Yuk, G. Tae and Y. H. Kim, *J Control Release*, 2010, **143**, 282–289.

32 Y. I. Chung, G. Tae and S. Hong Yuk, *Biomaterials*, 2006, **27**, 2621–2626.

33 S. M. Jay, B. R. Shepherd, J. W. Andrejecsk, T. R. Kyriakides, J. S. Pober and W. M. Saltzman, *Biomaterials*, 2010, **31**, 3054–3062.

34 Q. Tan, H. Tang, J. Hu, Y. Hu, X. Zhou, Y. Tao and Z. Wu, *Int J Nanomedicine*, 2011, **6**, 929–942.

35 A. Des Rieux, B. Ucakar, B. P. K. Mupendwa, D. Colau, O. Feron, P. Carmeliet and V. Préat, *J Control Release*, 2011, **150**, 272–278.

36 S. Noel, B. Liberelle, L. Robitaille, G. De Crescenzo, *Bioconjugate Chem*, 2011, **22**, 1690–1699.
